# Two Distinct Interferon-γ in the Orange-Spotted Grouper (*Epinephelus coioides*): Molecular Cloning, Functional Characterization, and Regulation in Toll-Like Receptor Pathway by Induction of miR-146a

**DOI:** 10.3389/fendo.2018.00041

**Published:** 2018-02-26

**Authors:** Wan Peng, Yan Sun, Gao-Fei Li, Liang-Ge He, Ruo-Zhu Li, Yao-Si Liang, Xu Ding, Xue Yu, Yong Zhang, Hao-Ran Lin, Dan-Qi Lu

**Affiliations:** ^1^State Key Laboratory of Biocontrol, Institute of Aquatic Economic Animals, Guangdong Provincial Key Laboratory for Aquatic Economic Animals, College of Life Sciences, Sun Yat-Sen University, Guangzhou, China; ^2^Sun Yat-Sen University Cancer Center, State Key Laboratory of Oncology in South China, Collaborative Innovation Center for Cancer Medicine, Guangzhou, China; ^3^Department of Experimental Research, Sun Yat-Sen University Cancer Center, Guangzhou, China

**Keywords:** *Epinephelus coioides*, IFNγ1, IFNγ2, miR-146a, TNF receptor-associated factor 6

## Abstract

Interferon gamma (IFNγ) is a Th1 cytokine that is critical for innate and adaptive immunity. Toll-like receptors (TLRs) signaling pathways are critical in early host defense against invading pathogens. miR-146a has been reported to participate in the regulation of host immunity. The known mechanisms of integrations between the IFNγ and TLR signaling pathways are incompletely understood, especially in teleosts. In this study, orange-spotted grouper (*Epinephelus coioides*) IFNγ1 and IFNγ2, their biological activities, especially their involvements in TLR pathway, were explored. We identified and cloned two IFNγ genes of *E. coioides*, namely *EcIFNγ1* and *EcIFNγ2*. The produced recombinant *E. coioides* IFNγ1 (r*Ec*IFNγ1) and IFNγ2 (r*Ec*IFNγ2) proteins showed functions, which are similar to those of other bony fishes, such as enhancing nitric oxide responses and respiratory burst response. r*Ec*IFNγ2 could regulate TLR pathway by enhancing the promoter activity of miR-146a upstream sequence and thus increasing the expression level of miR-146a, which possibly targets TNF receptor-associated factor 6 (TRAF6), a key adapter molecule in TLR signaling pathway. Taken together, these findings unravel a novel regulatory mechanism of anti-inflammatory response by IFNγ2, which could mediate TLR pathway through IFNγ2–miR-146a–TRAF6 negative regulation loop. It is suggested that IFNγ2 may provide a promising therapeutic, which may help to fine tune the immune response.

## Introduction

Interferon gamma (IFNγ) is a Th1 cytokine that is critical for almost all phases of immune and inflammatory responses. Only one single IFNγ gene is identified in mammalian (1), avian (2), and amphibian (3) species, while many fishes possess two IFNγ genes, namely IFNγ1 and IFNγ2. Like mammalian IFNγ, teleost IFNγs mediate their protective effects as an activator of macrophages, through the enhancing of respiratory burst activity, nitric oxide production, and bacterial phagocytosis (4–8), inducing of the expression of pro-inflammatory cytokines (4, 5, 9) and the typical antiviral genes (8, 10). Respiratory burst and nitric oxide are well known as potent antimicrobials (11).

Orange-spotted grouper (*Epinephelus coioides*) is one of the most commercially important species in Southeast Asian. However, along with the rapid development of aquaculture industry, diseases caused by viruses, bacteria, and parasites emerge more and more frequently and have led to great number of economic loss. Injection of Poly(I:C), an interferon inducer, or recombinant IFNa1 protein could provide significant protection against viruses in sevenband grouper (*Epinephelus septemfasciatus*) (12, 13). In addition, inactivated Singapore grouper iridovirus vaccine, which showed high efficiency in orange-spotted grouper, could induce the expression of type I interferon-stimulated genes, suggesting that type I interferon system may be involved in the antivirus immune responses (14). Taken together, it was suggested that interferons might play important roles in the immune system of groupers, while the function of IFNγ and its potential applications in grouper farming are still unclear and worth investigating.

Toll-like receptors (TLRs), a family of evolutionarily conserved receptors, play crucial roles in early host defense against invading pathogens. IFNγ was produced by natural killer and T cells during the recognition of pathogens by TLRs (15). Like mammalian IFNγ (16), teleost IFNγs also play various roles in response to bacterial or viral infection (8, 9, 17, 18). IFNγ and TLR signaling pathways are important for both innate and adaptive immune responses. However, the cross talk between IFNγ and TLR signal pathways is incompletely understood.

MicroRNAs (miRNAs) are conserved small non-coding RNAs that function as posttranscriptional regulators of gene expression by binding to the 3ʹ untranslated region (UTR) of target mRNAs (19–22). The genesis of miRNAs was mediated by a two-step processing pathway, in which long primary miRNAs are first processed to approximately 60-bp hairpin precursor miRNAs (pre-miRNAs), then, these pre-miRNAs are cleaved to generate mature miRNAs (23, 24). miR-146a has been reported to participate in the regulation of host immunity (25–28). miR-146a, whose transcription is controlled by NF-κB and is induced by TLR activation, negatively regulates the TLR pathway by targeting TNF receptor-associated factor 6 (TRAF6) and interleukin-1 receptor-associated kinase 1 (IRAK1), which are key adapter molecules in TLR signaling cascades, mediating activation of NF-κB pathway (29–34).

In the present study, we report the cloning, expression profiles of *E. coioides* IFNγ1 and IFNγ2 and their potential functions in regulation of immune response. First, the functional recombinant *E. coioides* IFNγ1 (r*Ec*IFNγ1) and recombinant *E. coioides* IFNγ2 (r*Ec*IFNγ2) proteins were obtained. We detected their ability of enhancing respiratory burst activity, nitric oxide production. Then the potential cross talk between *E. coioides* IFNγs and TLR pathway was explored. Our data demonstrated that r*Ec*IFNγ2 could regulate TLR pathway by enhancing the miR-146a upstream sequence transcription activity and thus increasing miR-146a expression, while miR-146a may target TRAF6. These findings unravel a novel regulatory mechanism of anti-inflammatory response by IFNγ2, which may function as novel negative regulator and promising therapeutic that help to fine tune the immune response.

## Materials and Methods

### Ethics Statement

All animal experiments were conducted in accordance with the guidelines and approval of the Animal Research and Ethics Committees of Sun Yat-Sen University. All efforts were made to minimize suffering.

### Fish

Healthy *E. coioides* weighing approximately 500 g were purchased from the Guangdong Daya Bay Fishery Development Center (Huizhou, Guangdong, P. R. China). The fish were maintained in a recirculating seawater system, with a 12 h light/12 h dark cycle, at 25–30°C for 7 days before use. The fish were fed with commercial pellets twice daily. Food was withheld from the fish for 24 h prior to sample collection. All of the fish used in this study appeared to be healthy before the experiments were conducted. Before sample collection, the fish were anesthetized with MS-222 in dechlorinated water for 2 min.

### Molecular Cloning

The head kidney was dissected from healthy *E. coioides*, and total RNA was extracted by TRIzol reagent (Invitrogen, USA). The first strand of cDNA was synthesized with ReverTra Ace qPCR RT Kit (TOYOBO, Japan) according to the manufacturer’s instructions.

The open reading frame (ORF) regions of *IFN*γ*1* and *IFN*γ*2* were amplified with the primers listed in Table 1. PCR amplification were performed at 94°C for 5 min, followed by 40 cycles at 94°C for 20 s, 55°C for 20 s, and 72°C for 45 s, with 72°C for a final 10 min at the end of the last cycle. All PCR products were ligated into the pTZ57R/T Vector (Fermentas, USA) after analyzing on 1.5% agarose gels, finally sequenced by Invitrogen Bioengineering Corporation, Guangdong, China.

**Table 1:** List of primer sequences.

Primers	Sequence 5′–3′	Information
IFNγ1 open reading frame (ORF)-F	ATGTCTTCGTGTTGTGGATC	IFNγ1 ORF cloning
IFNγ1 ORF-R	AGAAACAGTTCCCAGCACC	
IFNγ2 ORF-F	TGTCTCCGTCCTGAGCATC	IFNγ2 ORF cloning
IFNγ2 ORF-R	AGATTTGTCATTAAACACCCTC	
IFNγ1 NdeI-F	GGAATTCCATATGTCTAGGATTCCATG	Recombinant construct
IFNγ1 XhoI-R	CCGCTCGAGGCGTCGTTCAGCAGA	
IFNγ2 EcoRI-F	CCGGAATTCGTCCCACATCCCTCAGGAGAT	Recombinant construct
IFNγ2 XhoI-R	CCGCTCGAGGGCTCTCTGATGAGTTTTGA	
IFNγ1 FD	CGATTCGGTCATCAAGAGCAT	Real-time PCR
IFNγ1 RD	CTCCGTCACGACCGACACCA	
IFNγ2 FD	CAGCAATGGTGAGGTGGCA	
IFNγ2 RD	TTTGCTCTGGATGATAGGGTC	
18S F	CCTGAGAAACGGCTACCACATCC	
18S R	AGCAACTTTAGTATACGCTATTGGAG	
TNF receptor-associated factor 6 (TRAF6) F	GGTGGCAGTAACATGGCAAG	TRAF6 3′untranslated region cloning
TRAF6 R	GCTATTTTGTGTCAGTGTTGTCTCA	
miR-146a F	TTGAATACACCCCTATCAGACATC	The upstream of mature miR-146a cloning
miR-146a R	AGTAAGGCTGACAAACAAGTACCA	

The upstream sequence of pre-miR-146a was cloned from *E. coioides* genomic DNA using the primers of miR-146a F/R (Table 1). The pre-miR-146a upstream fragment was ligated into pGL4.10[*luc2*] vector (Promega, USA). Mutated versions of these constructs were obtained by site-directed mutagenesis using Mut Express II Fast Mutagenesis Kit (Vazyme, China).

TNF receptor-associated factor 6 3ʹUTR was cloned from head kidney cDNA by PCR with the primers of TRAF6 F/R (Table 1). To create 3ʹUTR luciferase reporter constructs, fragments of 3ʹUTR of TRAF6 gene were sub-cloned downstream of CMV-driven firefly luciferase cassette in pMIR-REPORT vector (Ambion, USA).

### Bioinformatics

BLAST was used for identifying cDNA and deducing amino acid sequences in the NCBI.^1^ Prediction of ORF were performed in the DNAssist 2.0 software. Multiple-sequence alignment of the *Ec*IFNγ with other vertebrate IFNγs were performed with the Clustal Omega.^2^ A vertebrata IFNγ phylogenetic tree was constructed with MEGA6 software using the maximum likelihood (ML) method, with a bootstrap of 100 times to verify its credibility. The SignalP program^3^ was applied to search for the signal peptide of vertebrata IFNγ. *N*-glycosylation sites were predicted by NetNGlyc^4^ and nuclear localization sequences (NLS) were predicted by Brameier et al. (35). Target prediction between miR-146a and the 3ʹUTR region of TRAF6 was performed using FINDTAR3. Sequences of mature miR-146a were obtained from our lab by whole genome sequencing. The pre-miR-14a was confirmed by structure prediction using *RNAfold* WebServer.^5^

### Expression Study

Twelve tissues including the thymus, head kidney, trunk kidney, spleen, heart, gill, eye, skin, intestine, stomach, skin, and liver were aseptically dissected from three independent individuals. The tissue expression profiles were detected by real-time PCR performed with SYBR Green PCR Master Mix (Life Technologies, USA) and the primers of IFNγ1 F/R or IFNγ2 F/R, respectively. *E. coioides* 18S rRNA (18S-F/R) was amplified as an internal control (Table 1).

### Production and Purification of r*Ec*IFNγ Proteins

The putative mature peptides of *E. coioides* IFNγ1 and IFNγ2 were predicted by the SignalP program. Then, the cDNA fragments encoding the putative mature peptide with deletion of the the signal peptide from the N terminus were amplified by PCR using the primers of IFNγ1 NdeI-F/IFNγ1 XhoI-R and IFNγ2 EcoRI-F/IFNγ2 XhoI-R (Table 1), respectively. The fragments were separated on a 1.5% agarose gel and purified by Qiagen gel extraction kit (Qiagen, Germany). After subcloning into pTZ57R/T vector for sequencing, the fragments were digested with restriction enzymes and then inserted into the pET22b expression vector (Novagen, USA). The *E. coli* BL21 (DE3) cells transformed with IFNγ1 recombinant construct and *E. coli* Rosetta (DE3) cells transformed with IFNγ2 recombinant construct were induced by different concentrations of IPTG. The cells were collected by centrifugation and the resultant recombinant proteins named r*Ec*IFNγ1 and r*Ec*IFNγ2 were purified using His·Bind Column (Novagen) according to the manufacturer’s protocol. The purity of r*Ec*IFNγs was checked on SDS-PAGE gel stained with coomassie brilliant blue R-250 (Sigma, USA), and the size of target proteins was measured by comparing the protein band location with a standard protein (Fermentas). Both proteins were detected by western blotting using a primary anti-His-tag monoclonal antibody (Novagen) and a secondary goat anti-mouse IgG (Amersham Biosciences, UK).

### Isolation of Blood Lymphocytes and Head Kidney Monocytes

The whole blood of fish was directly obtained from three fish (*n* = 3) using 5 mL syringe. Blood lymphocytes were isolated from whole blood using lymphocyte separation medium. The primary head kidney cells were obtained as previously described (36). Head kidney monocytes were isolated from primary head kidney cells using monocyte isolating kit (TBDscience, China). The isolated cells were washed and enumerated on a hemocytometer with trypan blue and resuspended at a concentration of 10^6^ cells/mL in tissue culture medium (TCM). The TCM was prepared from RPMI-1640 medium by adding 10% fetal bovine serum (Life Technologies), 2 mM l-glutamine (Sigma), and penicillin/streptomycin (Sigma).

### Nitric Oxide Assay

Primary blood lymphocytes were distributed into 96-well plates at a density of 10^6^ cells/mL. r*Ec*IFNγ1 or r*Ec*IFNγ2 were respectively added to the culture medium to reach a working concentration of 1, 10, and 100 ng/mL, and the control group was treated with TCM. These cells were incubated at 28°C in 5% CO_2_ for 72 h. Nitrite production was determined based on the Griess reaction with a NO determination kit (Beyotime Institute of Biotechnology, China).

### Respiratory Burst Assay

Primary blood lymphocytes cultivation and *in vitro* stimulation were performed as described in Nitric oxide assay. The respiratory burst assay was performed as previously described (8). These cells were incubated at 28°C in 5% CO_2_ for 18 h. Then, NBT (2 mg/mL, Sigma) and PMA (final concentration, 100 ng/mL, Sigma) were added to the cell cultures at room temperature. Absolute methanol was applied to fix the pelleted cells, and 70% methanol was applied to remove the non-reduced NBT. After air drying, the reduced NBT was dissolved using 2 M KOH and the blue crystals in the cytoplasm were dissolved by DMSO. Finally, the OD values were detected at 630 nm.

### Western Blot

Primary head kidney cells were distributed into 6-well plates at a density of 10^6^ cells/mL. r*Ec*IFNγ1 or r*Ec*IFNγ2 were, respectively, added to the culture medium to reach a working concentration of 1, 10, and 100 ng/mL, and the control group was treated with TCM. After incubation for 3 h at 28°C in 5% CO_2_, these cells were washed with PBS and lysed in a lysis buffer (Beyotime), which contained protease inhibitors (Sigma) and phosphatase inhibitors (Sigma). The protein lysates were separated by SDS-PAGE, then transferred onto nitrocellulose membranes. The membranes were blocked in 5% BSA in TBST for 1 h at room temperature followed by incubations with primary antibodies and the relevant HRP-conjugated secondary antibodies. The membranes were processed for ECL Western Blotting Detection Reagents (Pierce). Meanwhile, the TRAF6 expression levels in primary head monocytes after r*Ec*IFNγ1 or r*Ec*IFNγ2 treatment were also detected by western blot. Antibodies used in the study were TRAF6 (Santa Cruz, CA, USA) and β-actin (Proteintech, USA). The bands were analyzed semiquantitively by densitometry (grayscale analysis) with ImageJ software and normalized to their controls.

### miR-146a Expression Levels Detection

After treatment with r*Ec*IFNγ1 or r*Ec*IFNγ2, head kidney monocytes were collected. Total RNA was extracted by TRIzol reagent (Invitrogen), then reverse-transcribed and amplified with the Hairpin-it miRNAs RT-PCR Quantitation kit (GenePharma, China). *E. coioides* U6 snRNA served as control. The relative level of miR-146a expression was calculated using the comparative threshold (2^−ΔΔCt^) method (37).

### Detection of the Relationship between miR-146a and TRAF6

In order to confirm whether miR-146a could directly target the mRNA of TRAF6, HEK-293T cells were transfected with 40 nM miR-146a mimics (GenePharma) or negative control (nc) (GenePharma) using Lipofectamine 2000 (Invitrogen) according to the manufacturer’s protocol. 24 h after transfection, luciferase activity in 293T cells was measured using the Dual-Luciferase Reporter Assay System (Promega) according to the manufacturer’s instructions. The luciferase data were normalized by dividing the firefly luciferase activity by the activity of Renilla luciferase.

Primary head kidney monocytes were also transfected with miR-146a mimics or nc. The cells were lysed and the TRAF6 protein levels in the lysates were detected with western-blot.

### Pre-miR-146a Upstream Sequence Activity Assay

Mutant or wild-type pre-miR-146a upstream-reporter vectors were transfected into 293T cell using Lipofectamine 2000. After 6 h, the cells were stimulated with r*Ec*IFNγ1 or r*Ec*IFNγ2 and luciferase activities were measured.

### Statistical Analyses

All data were expressed as mean values ± SEM. Statistical analysis was carried out by one-way analysis of variance (ANOVA) or *t*-test. Differences were considered significant with a *p*-value less than 0.05 and were marked with asterisks.

## Results

### Cloning and Sequence Characterization of *Ec*IFNγ1 and *Ec*IFNγ2 Genes

**Figure 1:**
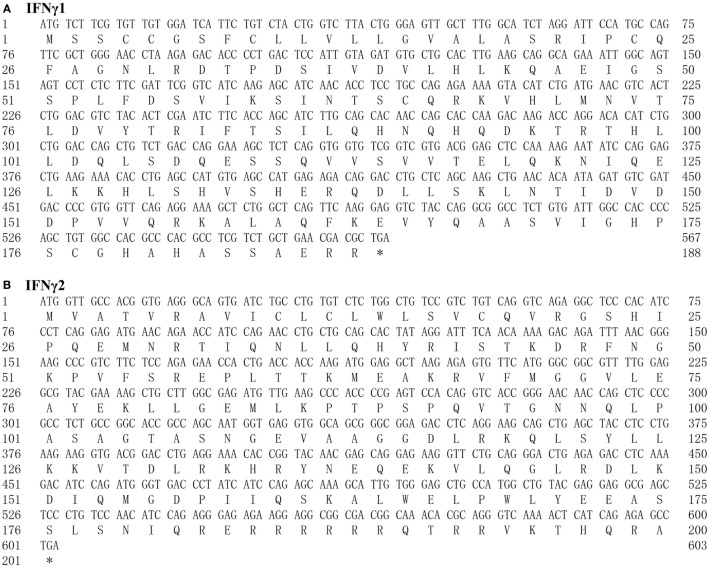
The nucleotide and predicted peptide sequences of *Epinephelus coioides* IFNγ1 **(A)** and IFNγ2 **(B)**. Start (ATG) and stop (TAG) codons are indicated in boldface type. The predicted signal sequence is underlined and glycosylation sites are double underlined. IFNγ signature sequence is boxed. The nuclear localization sequence (RRRR) is bolded.

The ORF of the *Ec*IFNγ1 and *Ec*IFNγ2 genes transcript was obtained (Figure 1). *Ec*IFNγ1 ORF was 567 bp in length and translated into a 188-aa precursor molecule with a 19-aa signal peptide, while *Ec*IFNγ2 ORF was 603 bp in length and encoded a 200-aa putative protein with a 19-aa signal peptide. The predicted mature *Ec*IFNγ1 peptides contain two potential *N*-glycosylation sites (NTS and NVT), while *Ec*IFNγ2 have one *N*-glycosylation site (NRT). Furthermore, *Ec*IFNγ1 and *Ec*IFNγ2 both possess an IFNγ signature sequence ([I/V]-Q-X-[K/Q]-A-X2-E-[L/F]-X2-[I/V]) at the C-terminus, which was conserved among known IFNγ molecules. In particular, a conserved motif RRRRRR similar to the nuclear localization signal of the known IFNγ molecules is found in *Ec*IFNγ2 at its C-terminal tail, which was absent in *Ec*IFNγ1.

### Homology Alignment and Phylogenetic Analysis

**Figure 2:**
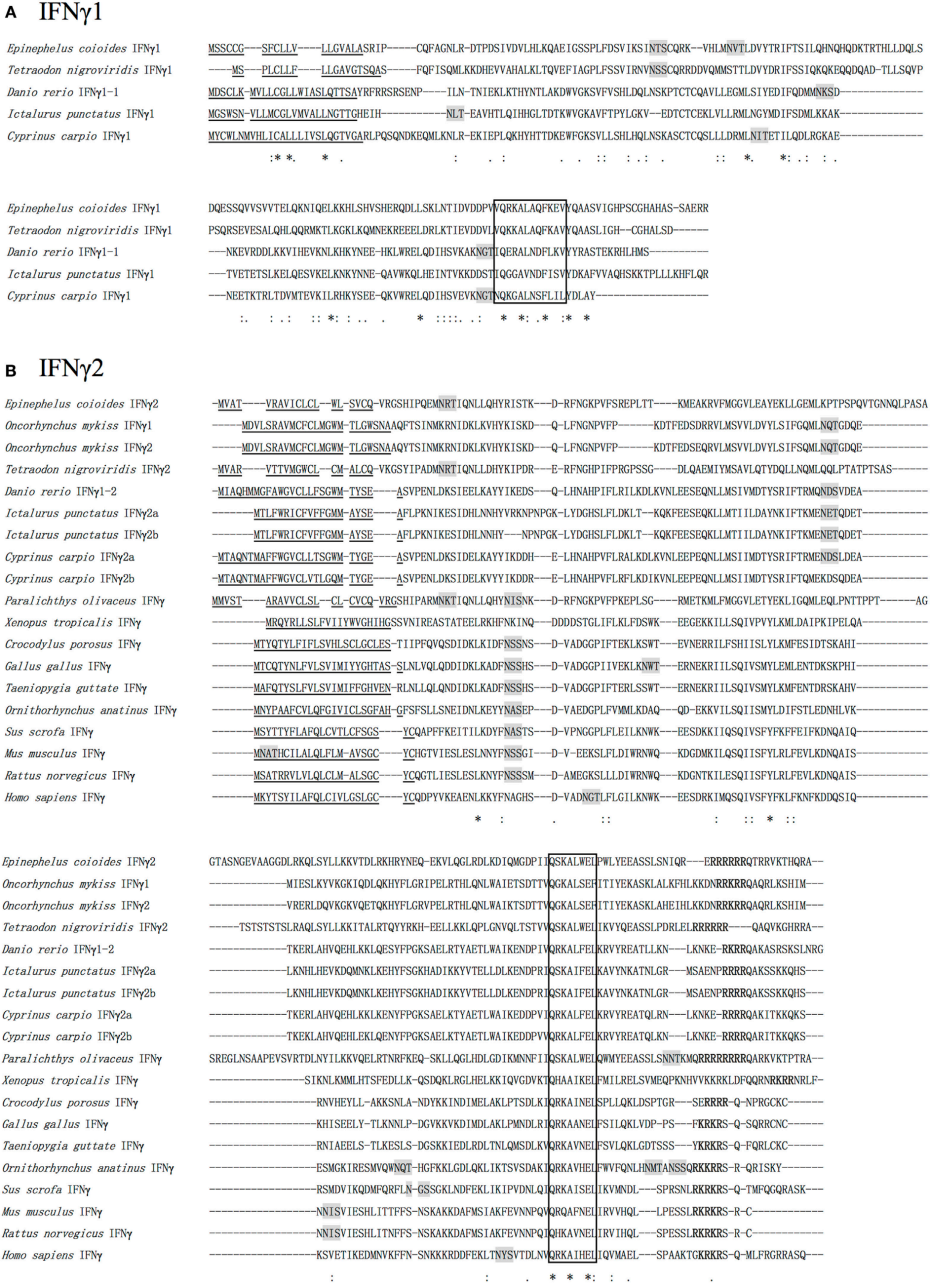
Multiple alignments of *Epinephelus coioides* IFNγ1 **(A)** and IFNγ2 **(B)**. A multiple alignment of the deduced amino acid sequences of *E. coioides* IFNγ1 and IFNγ2 with those of other mammalian and piscine sequences was created using the ClustalX program. The putative signal peptides are indicated by single underlines. The signature motif ([IV]-Q-X-[KQ]-A-X2-E-[LF]-X2-[IV]) is boxed. The *N*-glycosylation sites are shaded in gray. The nuclear localization sequences sequence is in bold. Identical amino acids among all sequences are indicated by “*,” whereas those with high or low similarity are indicated by “:” and “.”. Amino acids were collected form NCBI were listed as follows: NP_000610.2, *Homo sapiens* IFNγ; NP_032363.1, *Mus musculus* IFNγ; NP_620235.1, *Rattus norvegicus* IFNγ; NP_990480.1, *Gallus gallus* IFNγ; XP_002188959.1, *Taeniopygia guttata* IFNγ; NP_999113.1, *Sus scrofa* IFNγ; XP_007664509.1, *Ornithorhynchus anatinus* IFNγ; XP_019398803.1, *Crocodylus porosus* IFNγ; XP_002938555.1, *Xenopus tropicalis* IFNγ; NP_001153975.1, *Oncorhynchus mykiss* IFNγ1; NP_001153976.1, *O. mykiss* IFNγ2; BAD72865.1, *Danio rerio* IFNγ1-1; NP_998029.1, *Danio rerio* IFNγ1-2; AHZ62713.1, *Tetraodon nigroviridis* IFNγ1; AHZ62714.1, *T. nigroviridis* IFNγ2; BAG50577.1, *Paralichthys olivaceus* IFNγ; CAJ98867.1, *Cyprinus carpio* IFNγ1; CAJ51089.1, *C. carpio* IFNγ2b; CAJ51088.1, *C. arpio* IFNγ2a; AAZ40504.1, *Ictalurus punctatus* IFNγ1; AAZ40505.1, *I. punctatus* IFNγ2a; AAZ40506.1, *I. punctatus* IFNγ2b.

Both *Ec*IFNγ1 and *Ec*IFNγ2 sequences and the IFNγs from other species were subjected to multiple alignment (Figure 2). The alignment confirms that *Ec*IFNγ1 and *Ec*IFNγ2 have relatively low sequence similarity compared with their vertebrate counterparts, respectively. *Ec*IFNγ1 has 18.42–44.89% amino acid identity with other known fish IFNγ1, which shared the highest degree with tetraodon (*Tetraodon nigroviridis*) (44.89%). *Ec*IFNγ2 shared a higher similarity with teleosts IFNγ2 sequences varied from 25.15 to 61.86% than with other vertebrates (12.41–22.02%), being most similar to IFNγ from Japanese flounder (*Paralichthys olivaceus*) (61.86%).

**Figure 3:**
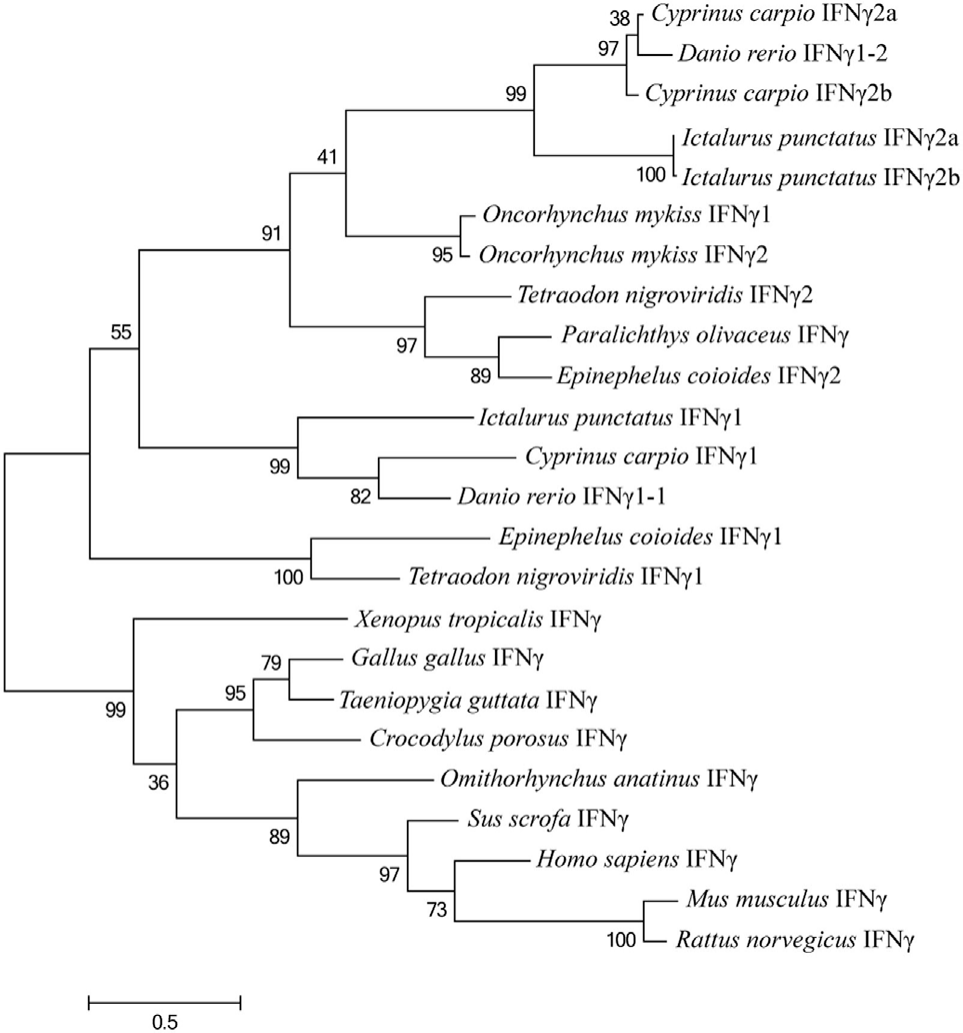
Phylogenetic tree, comparing the amino acid sequences of vertebrate interferon gamma (IFNγ) genes. This tree was generated with MEGA6 software using the maximum likelihood method, with a bootstrap of 100 times to verify its credibility. Values in percentage are indicated at branch nodes. All the amino acids sequences have already been applied in the multiple alignments in Figure 2.

A phylogenetic tree was also constructed based on the alignments of *Ec*IFNγ1 and *Ec*IFNγ2 sequences with that of other species (Figure 3). The *Ec*IFNγ1 was branched with *T. nigroviridis*. *Ec*IFNγ2 was most closely related to those of *P. olivaceus* and *T. nigroviridis*, all of which belonged to marine teleost IFNγ2 clade. The previously reported rainbow trout (*Oncorhynchus mykiss*) IFNγ1 might also belong to the IFNγ2 family based on the phylogenetic tree and protein alignments.

### Expression Profile of *Ec*IFNγ1 and *Ec*IFNγ2

**Figure 4:**
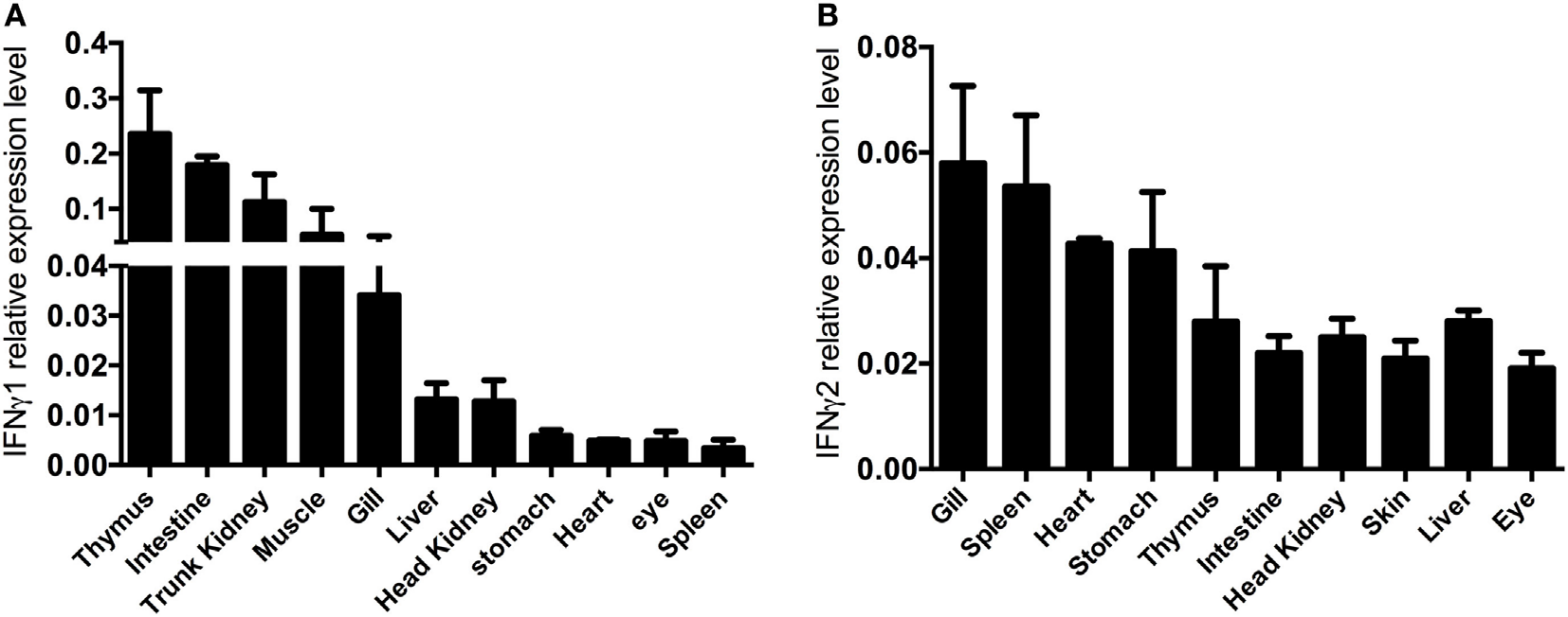
Constitutive interferon gamma (IFNγ) expression. cDNA of different organs of four control fish were used as template for quantitative real-time PCR. The IFNγ1 **(A)** and IFNγ2 **(B)** mRNA expression data are shown relative to the 18S rRNA.

Quantitative expression analysis of *Ec*IFNγ1 or *Ec*IFNγ2 in tissues of healthy fish revealed that *Ec*IFNγ1 and *Ec*IFNγ2 mRNA were expressed in different patterns. The highest mRNA levels of *Ec*IFNγ1 were in the thymus and intestine, while the expression was relatively low in stomach, heart, eye, and spleen (Figure 4A). *Ec*IFNγ2 was globally expressed in almost all tissues, and its expression levels were higher in gills and spleen, but lower in intestine, head kidney and eye (Figure 4B).

### Prokaryotic Production of rIFNγ1 and rIFNγ2

**Figure 5:**
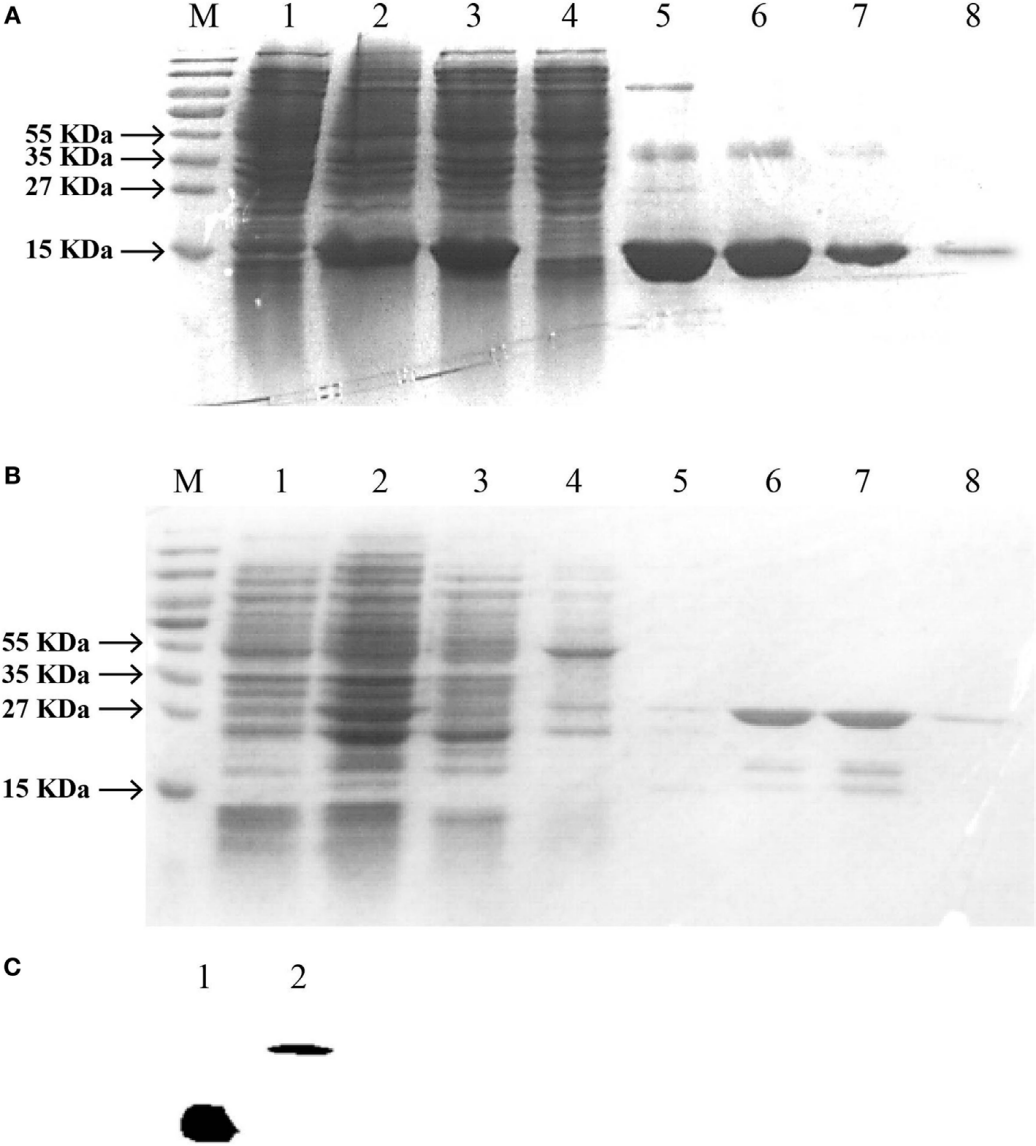
SDS-PAGE and western blotting analysis of *Epinephelus coioides* interferon gamma (IFNγ). IFNγ1 **(A)** or IFNγ2 **(B)** proteins was expressed using the pET22b expression vector. **(A)** Lane M: protein size marker; Lane 1: the total lysates from non-induced cells; Lane 2: the total lysates from IPTG-induced cells; Lane 3: soluble IFNγ1 protein; Lane 4–7: IFNγ1 protein by washing buffer with different imidazole concentration; Lane 8: purified recombinant protein with a His-tag; **(B)** Lane M: protein size marker; Lane 1: the total lysates from non-induced cells; Lane 2: the total lysates from IPTG-induced cells; Lane 3: soluble IFNγ2 protein; Lane 4–8: IFNγ2 protein by washing buffer with pH6.3, pH5.9, pH5.4, pH5.0, and pH4.5. **(C)** Verification of recombinant IFNγ1 and IFNγ2 by Western blot. Lane 1: IFNγ1; Lane 2: IFNγ2; they were analyzed on a 15% SDS-PAGE gel, followed by western blotting analysis using a mAb against the His-tag. The molecular weight of the lane 1 IFNγ1 was the same to that of the lane 8 of Figure 5A. Similarly, that of lane 2 IFNγ2 was also the same to the lane 8 of Figure 5B.

For further study of the biological activities of *Ec*IFNγ1 and *Ec*IFNγ2, the two putative mature peptides were expressed as C-terminal 6 His-tagged fusion protein in BL21 (DE3) and Rosetta (DE3) cells, respectively, followed by purification with affinity chromatography. SDS-PAGE analysis (Figures 5A,B) indicated that the purified sample exhibited a single protein band after induction by IPTG, and the molecular weights were close to the predicted size of the 6-His fused r*Ec*IFNγ1 and r*Ec*IFNγ2 peptides, respectively. Besides, both r*Ec*IFNγ1 and r*Ec*IFNγ2 were confirmed by Western blot assay with monoclonal antibody against the His-tag, and a single band was detected for the each of fused proteins (Figure 5C).

### r*Ec*IFNγ1 and r*Ec*IFNγ2 Activated Nitric Oxide Responses and Respiratory Burst Response

**Figure 6:**
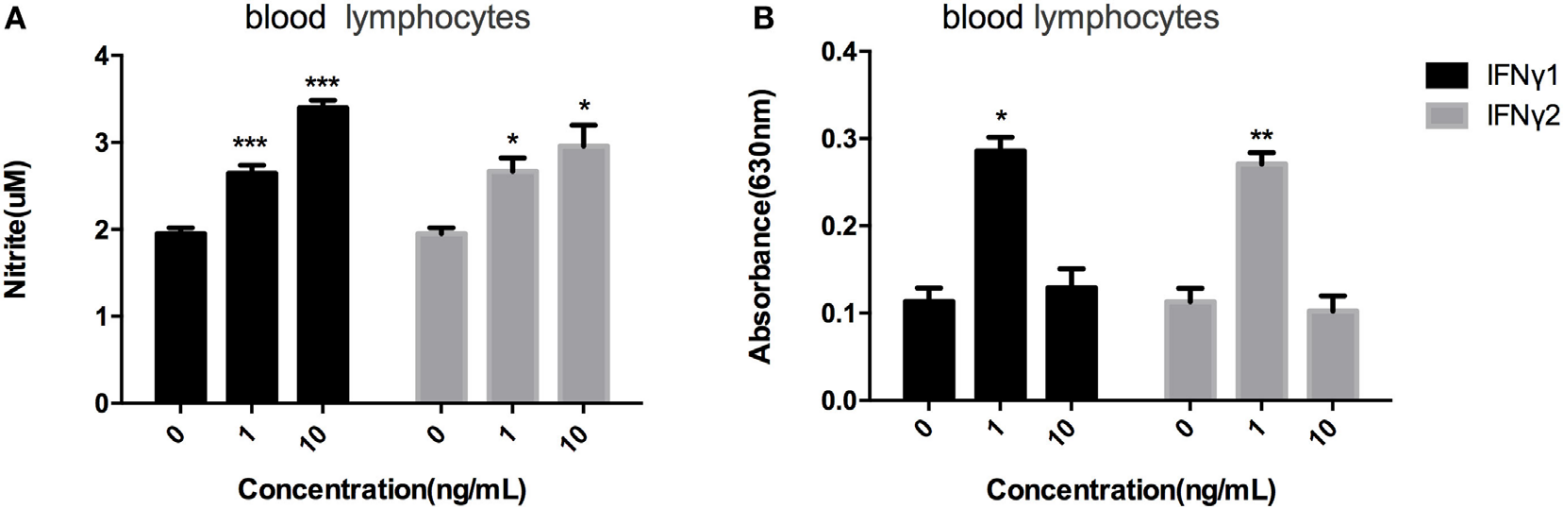
r*Ec*IFNγ1 and r*Ec*IFNγ2 activated nitric oxide responses and respiratory burst response. **(A)** Nitric oxide production of *Epinephelus coioides* primary blood lymphocytes after stimulation with various concentrations of rEcIFNγ1 or r*Ec*IFNγ2 for 72 h was determined using the Griess reaction and nitrite concentration was determined using a nitrite standard curve. **(B)** Respiratory burst activity of *E. coioides* primary blood lymphocytes after stimulation with various concentrations of r*Ec*IFNγ1 or r*Ec*IFNγ2 for 18 h was detected. Each bar indicates the mean ± SEM (*n* = 6). Statistical analysis was done using *t*-test. *, **, and ***: significant differences from control cells at the *p* < 0.05, *p* < 0.01, and *p* < 0.001 levels, respectively.

Blood lymphocytes were exposed to different concentrations of r*Ec*IFNγ1 or r*Ec*IFNγ2, followed by detection of the respiratory burst or nitric oxide levels. Both r*Ec*IFNγ1 and r*Ec*IFNγ2 at concentrations of 1 and 10 ng/mL induced nitric oxide responses in blood lymphocytes that were significantly higher than that of none recombinant protein treatment (Figure 6A). In addition, r*Ec*IFNγ1 or r*Ec*IFNγ2 at 1 ng/mL induced an obvious respiratory burst response in blood lymphocytes (Figure 6B).

### r*Ec*IFNγ1 and r*Ec*IFNγ2 Induced miR-146a Expression Level by Enhancing the miR-146a Upstream Region Activity

**Figure 7:**
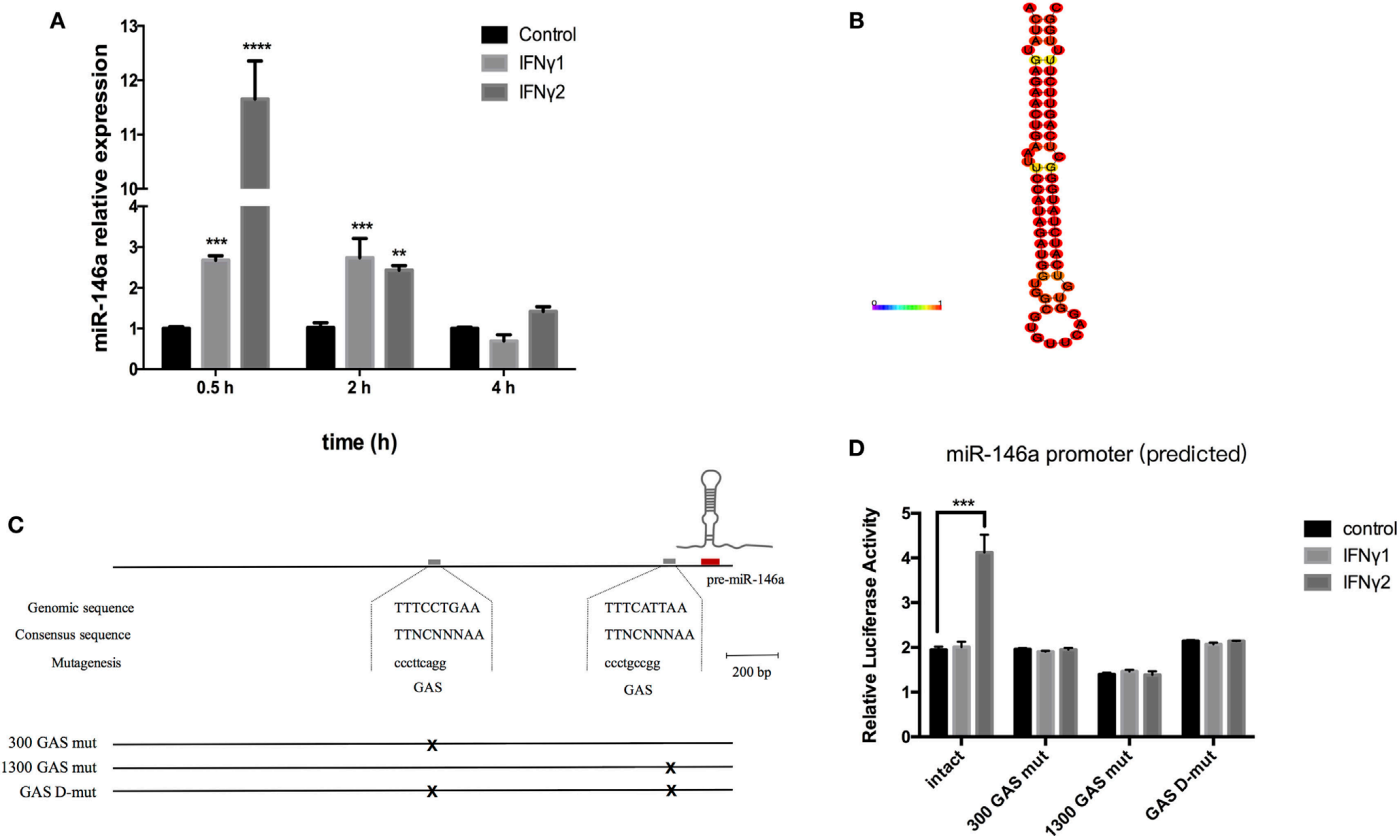
r*Ec*IFNγ1 and r*Ec*IFNγ2 induced the expression level of miR-146a. **(A)** Primary monocytes of head kidney were stimulated with 10 ng/mL r*Ec*IFNγ1 or r*Ec*IFNγ2 for different hours. The amount of miR-146a was normalized to that of U6 snRNA and was presented as the relative fold changes using the comparative threshold (2^−ΔΔCt^) method. **(B)** The stem loop of *Epinephelus coioides* pre-miR-146a was identified by RNA fold. **(C)** The approximately 2.5 kb genomic region upstream of mature miR-146a was analyzed. Two gamma-activated sequence (GAS, TTNCTTTAA) were found, namely 300 GAS and 1300 GAS. **(D)** The miR-146a upstream approximately 2.5 kb fragment was inserted into pGL4.10 vector. Dual-luciferase report assay was conducted after transfection with wild-type or GAS mutant pGL4-miR-146a vectors followed by r*Ec*IFNγ1 or r*Ec*IFNγ2 stimulation. The miR-146a upstream region luciferase activity significant increased after r*Ec*IFNγ2 treatment. Upon mutant of either GAS, r*Ec*IFNγ2 had no effect on luciferase activity of miR-146a upstream region. Each bar indicates the mean ± SEM (*n* = 6). Statistical analysis was performed using one-way analysis of variance. *, **, and ***: significant differences from control cells at the *p* < 0.05, *p* < 0.01, and *p* < 0.001 levels, respectively.

The miR-146a expression level was detected by real-time PCR. Compared to the control group, the expression level of miR-146a in group with r*Ec*IFNγ1 or r*Ec*IFNγ2 treatment was significantly increased at 0.5 and 2 h (Figure 7A). miR-146a expression level reached a peak (increased approximately 11-fold) at about 2 h after r*Ec*IFNγ2 treatment. The rapid induction of miR-146a in response to r*Ec*IFNγ1 or r*Ec*IFNγ2 suggested that miR-146a might be involved in response to IFNγ.

We first identified the stem loop of *E. coioides* pre-miR-146a (Figure 7B). Then, approximately 2.5 kb genomic region upstream of mature miR-146a was analyzed. Two gamma-activated sequence (GAS, TTNCTTTAA) were found, namely 300 GAS (−198 to −190 bp) and 1300 GAS (−1,136 to −1,128 bp) (Figure 7C). To explore the relationship between GAS and *E. coioides* IFNγs, the miR-146a upstream approximately 2.5 kb fragment was inserted into pGL4.10 vector. Dual-luciferase report assay was conducted after transfection with wild-type or mutant pGL4-miR-146a vectors followed by r*Ec*IFNγ1 or r*Ec*IFNγ2 stimulation. The miR-146a upstream region luciferase activity significant increased after r*Ec*IFNγ2 treatment (Figure 7D). Upon mutantion of either GAS, r*Ec*IFNγ2 had no effect on luciferase activity of miR-146a upstream region.

### miR-146a May Target TRAF6

**Figure 8:**
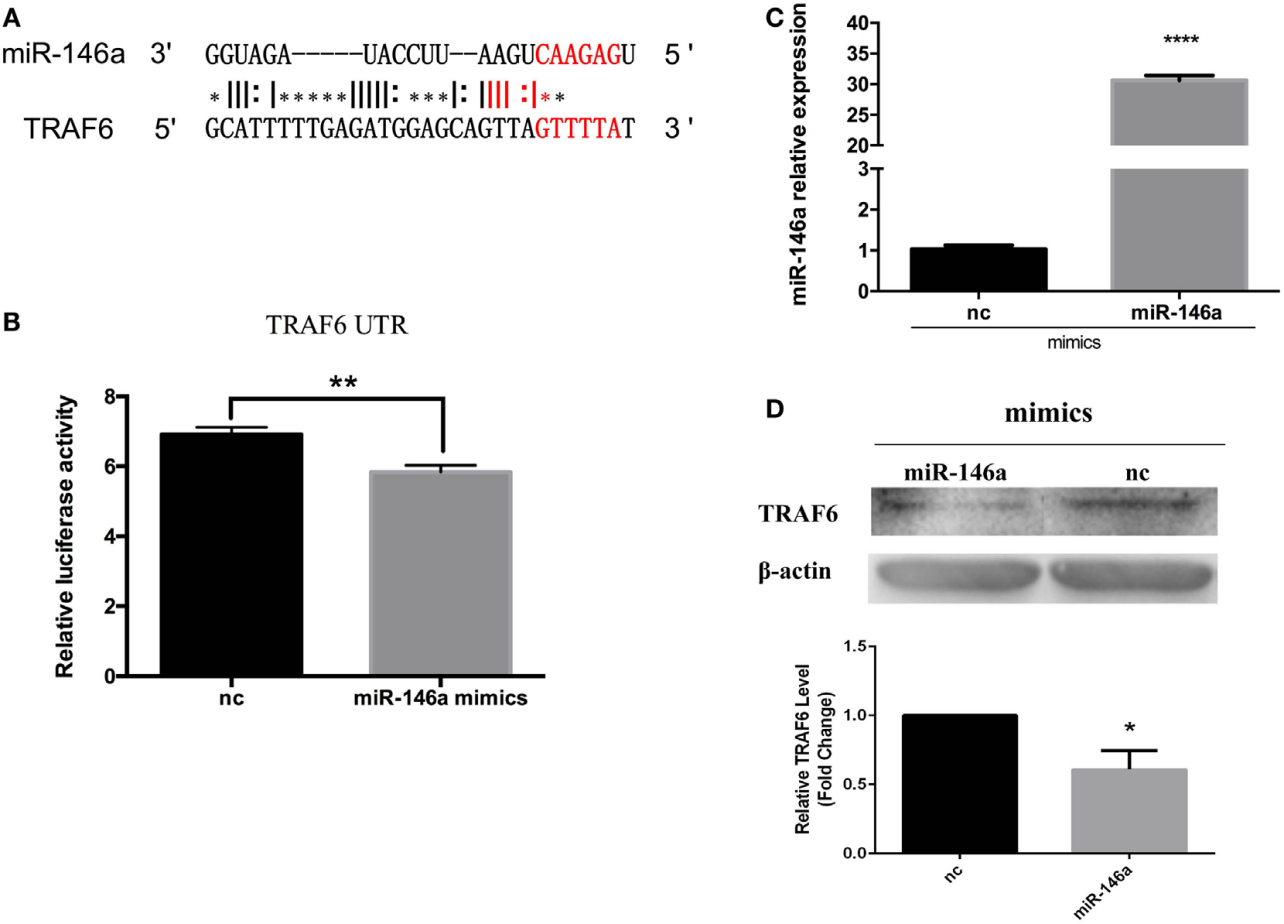
*Epinephelus coioides* miR-146a may target TNF receptor-associated factor 6 (TRAF6). **(A)** The target site in TRAF6 was predicted by FINDTAR3. **(B)** A reporter construct that contain the firefly luciferase gene fused to 900 bp of the 3ʹ untranslated region from TRAF6 containing putative miR-146 target site was obtained. miR-146a mimics induced the obvious downregulation of TRAF6 luciferase activity. **(C)** The *E. coioides* monocytes of head kidney were treated miR-146a mimics or negative control (nc). Then, the fold expression of miR-146a was calculated by the 2^−ΔΔCt^ method using U6 as the reference gene, respectively. Compared with the nc, the expression of miR-146a significantly increased after miR-146a mimic treatment. **(D)** Treatment with miR-146a mimics triggered the weakening of the TRAF6 protein. The quantified graph was shown below the blots. Each bar indicates the mean ± SEM (*n* = 6). Statistical analysis was performed using one-way analysis of variance. *, **, and ***: significant differences from control cells at the *p* < 0.05, *p* < 0.01, and *p* < 0.001 levels, respectively.

To further investigate the effect of miR-146a induction, we predicted its mRNA target sites. 3ʹUTR of TRAF6 mRNA was found to contain miR-146a target sequences (Figure 8A). In mammals, TRAF6 are key adapter molecules in TLR receptor signaling cascades, mediating activation of NF-κB pathway. To test the possibility that the TRAF6 expression may be regulated posttranscriptionally by miR-146a, we constructed a reporter vector that contained the firefly luciferase gene fused to 900 bp of the 3ʹUTR region of TRAF6 containing putative miR-146 target site. The reporter construct was transiently transfected into 293T cells together with miR-146a mimics. Compared to nc, a significant downregulative effect in the relative luciferase activity was observed when cells were transfected with miR-146a mimics (Figure 8B). We further examined the effect of miR-146a on TRAF6 protein expression in primary head kidney monocytes of *E. coioides*. After miR-146a mimic treatment, miR-146a mRNA expression was found to increase, which suggested that miR-146a mimics was successfully transfected into the primary head kidney monocytes (Figure 8C). The western blot results showed that TRAF6 protein level decreased after miR-146a mimics transfection (Figure 8D). These data suggested that the TRAF6 gene might be the target for posttranscriptional repression by miR-146a.

### r*Ec*IFNγ1 and r*Ec*IFNγ2 Regulated TRAF6 Expression

**Figure 9:**
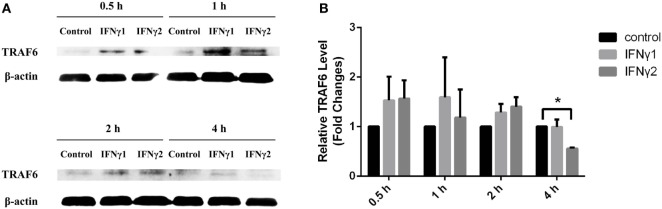
r*Ec*IFNγ1 and r*Ec*IFNγ2 regulated TNF receptor-associated factor 6 (TRAF6) expression. **(A)** Primary monocytes of head kidney were stimulated with 10 ng/mL r*Ec*IFNγ1 or r*Ec*IFNγ2 for different hours. The expression of TRAF6 protein was detected using western blot. The β-actin was used as an internal control. **(B)** Grayscale analysis was performed to semi-quantify the blots. *: significant difference from control blots at the *p* < 0.05 levels (*n* = 3).

Compared to the control group, both r*Ec*IFNγ1 and r*Ec*IFNγ2 treatments obviously induced TRAF6 protein expression from 0.5 to 2 h. After 4 h, r*Ec*IFNγ1 treatment slightly increased the TRAF6 protein expression, while r*Ec*IFNγ2 treatment resulted in slight decrease (Figure 9).

## Discussion

IFNγ1 and IFNγ2 are already identified in various teleosts. However, their immune functions, especially their involvement in TLR pathway, remained unclear. In this research, the biological activities of r*Ec*IFNγ1 and r*Ec*IFNγ2 were preliminarily characterized. We demonstrated that *E. coioides* IFNγ1 and IFNγ2 not only functioned similarly to those of other bony fishes but could also mediate TLR pathway through IFNγ2-miR-146a-TRAF6 negative regulation loop.

*Ec*IFNγ1 shares highest sequence similarity with *T. nigroviridis* (44.89%) IFNγ1 molecules and lower similarity with IFNγ2 proteins. In bony fish, IFNγ2 shows higher sequence similarity with marine fishes generally than freshwater fishes and shares highest identity with *P. olivaceus* (61.86%). Based on the relatively low similarity between species, it might be suggested that IFNγs of different species play different roles. However, the protein tertiary structure of the whole IFNγs family as six α-helices remained conserved. Besides, a conserved motif ([IV]-Q-X-[KQ]-A-X2-E-[LF]-X2-[IV]) could be found in *Ec*IFNγ1, *Ec*IFNγ2 and all the other known IFNγs. The C-terminal cationic motif RRRRRR is found in *Ec*IFNγ2, which is similar to its mammalian counterparts. This motif is considered as the NLS region that plays indispensable role in IFNγ functioning. Loss of NLS resulted in a failure of the inducing ability of rIFNγ on IP-10 expression in *O. mykiss* (7).

*Ec*IFNγ1 and *Ec*IFNγ2 present ubiquitous and constitutive expression in many tissues of teleost fish but show different expression patterns. In Atlantic cod (*Gadus morhua*) and Atlantic halibut (*Hippoglossus hippoglossus L*.), IFNγ2 is mainly expressed in gills and spleen, while its expression levels are low in stomach and liver (18, 38). In common carp (*Cyprinus carpio L*.) and goldfish, IFNγ2 is ubiquitously expressed in almost all tissues, while the highest expression levels could be found in immune organs as well (5, 39). In channel catfish (*Ictalurus punctatus*), IFNγ1 was found to be highly expressed in the thymus and intestines, while IFNγ2 was found to be only expressed in head kidney (40). In contrast, in rainbow trout, IFNγ1 mRNA transcription profile is similar to IFNγ2, both of whom were highly expressed in gills and spleen, with lower expression in head kidney and skin (7), which further suggested that the rainbow trout IFNγ1 might belong to IFNγ2 family.

In this study, *Ec*IFNγ1 and *Ec*IFNγ2 gene showed broad expression in all sampled tissues. *Ec*IFNγ1 was expressed at highest level in thymus and intestines while *Ec*IFNγ2 in spleen and gills, suggesting the two IFNγ genes might participate in signaling pathways of both immune and in non-immune tissues to resist pathogens in different manner probably.

Although recombinant proteins produced from *E. coli* generally lack glycosylation, whether the functions of interferons rely on glycosylation or not remains unclear. In black carp, it was reported that the un-glycosylated mutation form of IFNα could still be secreted and showed the similar antiviral ability as that of normal IFNα (41). For IFNγ, it was found that the unglycosylated IFNγ suffered from shorter half-life and lower protease resistance, while its function seemed to be unchanged (42). Functional *in vitro* assays exhibited that r*Ec*IFNγ1 and r*Ec*IFNγ2 could induce nitric oxide response, which effects are similar with the previous researches from mammals, goldfish, tetraodon, and common carp, in which, IFNγ were found to induce a nitric oxide response (5, 6, 8, 39, 43). Meanwhile, both r*Ec*IFNγ1 and r*Ec*IFNγ2 significantly enhanced the respiratory burst response, which effects were also observed in mammalian, goldfish and rainbow trout IFNγs (5–7, 44). These results suggested that the rEcIFNγ1 and rEcIFNγ2 obtained from *E. coli* have normal biological activities similar to those of the type II interferons of other teleosts.

After r*Ec*IFNγ1 or r*Ec*IFNγ2 treatment, the expression of TRAF6 protein was first upregulated, which lasted for 2 h and then gradually decreased to the basic level. It suggested that there might also exist a cross talk between IFNγ and TLR signaling pathway in teleost. In mammals, IFNγ regulates TLR signaling components (45) by including positive TLR signaling components, such as TLR itself, signaling adaptors MyD88 and IRAK1 (46, 47), and inhibitory TLR signaling components, such as SOCS1 and SOCS3 (48). IFNγ also could modulate the expression of ICSBP expression, which may participate more directly in the TLR signaling pathway, *via* interaction with TRAF6 (49). Our results indicated a tight interplay between *E. coioides* IFNγs and TLR signaling pathways through induction of TRAF6 proteins.

miR-146a is one of the most important and well-characterized miRNAs (26, 27, 50) and is reported as a key regulator of the immune response through mediating TLR signaling. In mammals, the TLR4 signaling pathway can activate the expression of miR-146a mediated by NF-κB (30). Besides, miR-146a negatively regulates NF-κB activation through targeted inhibition of the signaling proteins of innate immune responses, such as NF-κB inducers TRAF6 and IRAK1 (51). In shellfish, miR-146a was identified in *Pinctada martensii* and represented a critical role in inflammatory response (52). In consistent with our results of the r*Ec*IFNγ1 and r*Ec*IFNγ2 induction of miR-146a expression, miR-146a expression level was also upregulated when A375 cells were stimulated with IFNγ in a short time (less than 12 h) (53). It raised a question whether IFNγ was involved in the regulation of miR-146a expression. What is more, the luciferase reporter assay further suggested that r*Ec*IFNγ2 enhanced miR-146a upstream sequence luciferase activity, which was mediated by GAS site in miR-146a upstream region. Meanwhile, as the findings of previous researches in mammals, *E. coioides* miR-146a may target TRAF6, which is an adaptor in TLR pathway. Thus, we proposed that an IFNγ2-miR-146a-TRAF6 negative loop may exist in teleost. In addition, overexpression of NF-κB p65 increased the miR-146a upstream sequence luciferase reporter activity, which is mediated by binding to NF-κB binding site in miR-146a upstream region (data not shown). These results also showed that the IFNγ2-induced transcription of miR-146a in *E. coioides* relied on NF-κB as that of mammals.

**Figure 10:**
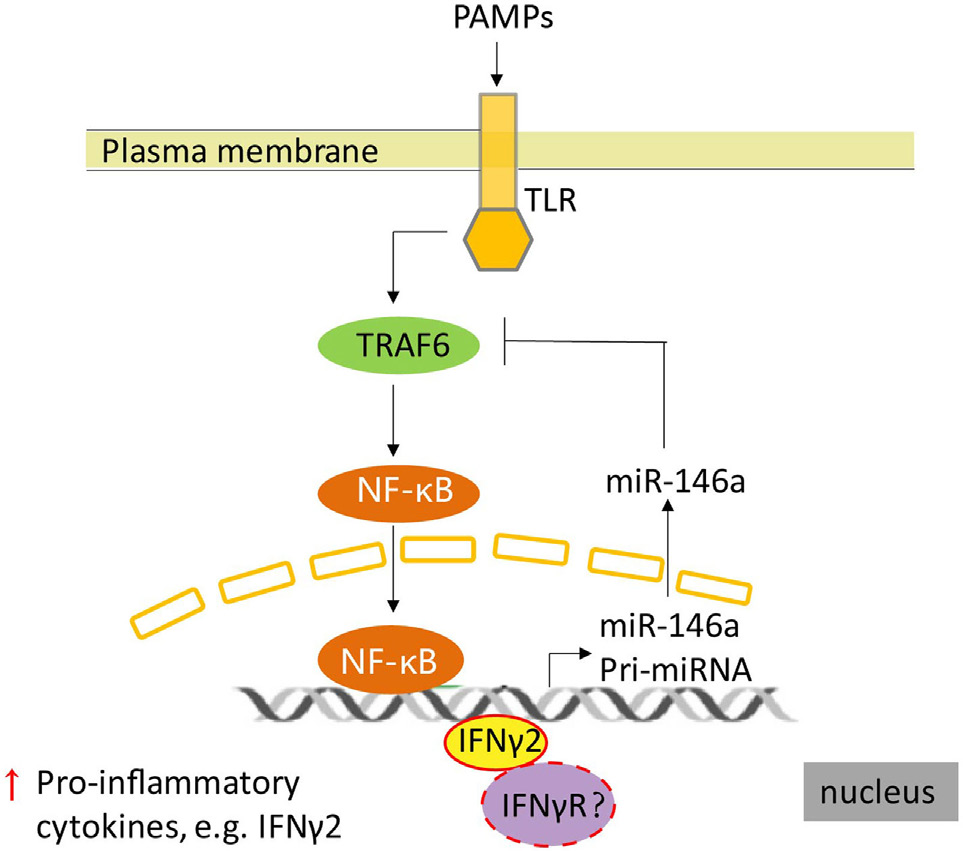
A supposed schematic of regulation between IFNγ2 and toll-like receptor (TLR) pathway in *Epinephelus coioides*. TLRs recognized the components of bacterial, then triggered the inflammatory response mediated by NF-κB signal, which induced the expression of pro-inflammatory factors, including IFNγ2. In *E. coioides*, subsequently, the expression of miR-146a was activated by NF-κB (the data were not shown) and IFNγ2. Upregulation of miR-146a reduced the expression of TNF receptor-associated factor 6, which is a signal transduction component in the TLR signaling pathway. Then the NF-κB signal was regulated. Meanwhile, how does *E. coioides* IFNγ2 activate the transcription of miR-146a and whether IFNγ receptor is involved in this regulation still remain unclear in *E. coioides*.

Our previous study in *T. nigroviridis* indicated that although *T. nigroviridis* IFNγs were not able to protect *T. nigroviridis* from *Vibrio parahaemolyticus* infection, *T. nigroviridis* IFNγ1 may promote an overwhelming inflammatory response, whereas *T. nigroviridis* IFNγ2 suppressed the inflammatory reaction promoted by *V. parahaemolyticus* infection (8). These further supported our speculation that r*Ec*IFNγ2 would regulate immune response by IFNγ2-miR-146a-TRAF6 negative regulation loop (Figure 10). A model of pathogen infection of *E. coioides* is needed to be established to further explore and characterize the *E. coioides* IFNγs function *in vivo* during immune response.

## Ethics Statement

All animal experiments were conducted in accordance with the guidelines and approval of the Animal Research and Ethics Committees of Sun Yat-Sen University. All efforts were made to minimize suffering.

## Author Contributions

WP and D-QL conceived and designed the experiments; WP and YS performed most of the experiments; L-GH, XY, and R-ZL performed some experiments; Y-SL and XD contributed reagents/materials. YZ and H-RL provided scientific advice; WP analyzed the data and wrote the manuscript. D-QL and G-FL revised and edited the manuscript. All authors reviewed the manuscript.

## Conflict of Interest Statement

The authors declare that the research was conducted in the absence of any commercial or financial relationships that could be construed as a potential conflict of interest.

## Footnotes

^1^https://blast.ncbi.nlm.nih.gov/Blast.cgi.

^2^http://www.ebi.ac.uk/Tools/msa/clustalo/.

^3^http://www.cbs.dtu.dk/services/SignalP/.

^4^http://www.cbs.dtu.dk/services/NetNGlyc/.

^5^http://rna.tbi.univie.ac.at/cgi-bin/RNAWebSuite/RNAfold.cgi.

## Abbreviations

IFNγ, interferon gamma; TLR, toll-like receptor; rEcIFNγ1, recombinant *E. coioides* IFNγ1; rEcIFNγ2, recombinant *E. coioides* IFNγ2; TRAF6, TNF receptor-associated factor 6; IRAK1, interleukin-1 receptor-associated kinase 1; UTR, untranslated region; ORF, open reading frame; NLS, nuclear localization sequences; TCM, tissue culture medium; PAMPs, pathogen-associated molecular patterns; IFNγR, IFNγ receptor.
